# The V471A Polymorphism in Autophagy-Related Gene *ATG7* Modifies Age at Onset Specifically in Italian Huntington Disease Patients

**DOI:** 10.1371/journal.pone.0068951

**Published:** 2013-07-22

**Authors:** Silke Metzger, Carolin Walter, Olaf Riess, Raymund A. C. Roos, Jørgen E. Nielsen, David Craufurd, REGISTRY Investigators of the European Huntington’s Disease Network, Huu Phuc Nguyen

**Affiliations:** 1 Institute of Medical Genetics and Applied Genomics, University of Tuebingen, Tuebingen, Germany; 2 Rare Disease Center, University of Tuebingen, Tuebingen, Germany; 3 Department of Neurology, Leiden University Medical Centre, Leiden, The Netherlands; 4 Memory Disorders Research Unit, Neurogenetics Clinic, Section 6702, Copenhagen University Hospital, Rigshospitalet, Copenhagen, Denmark; 5 Institute of Cellular and Molecular Medicine, Section of Neurogenetics, University of Copenhagen, The Panum Institute, Copenhagen, Denmark; 6 Genetic Medicine, University of Manchester, Manchester Academic Health Sciences Centre and Central Manchester University Hospitals NHS Foundation Trust, St. Mary’s Hospital, Manchester, United Kingdom; Emory University, United States of America

## Abstract

The cause of Huntington disease (HD) is a polyglutamine repeat expansion of more than 36 units in the huntingtin protein, which is inversely correlated with the age at onset of the disease. However, additional genetic factors are believed to modify the course and the age at onset of HD. Recently, we identified the V471A polymorphism in the autophagy-related gene *ATG7*, a key component of the autophagy pathway that plays an important role in HD pathogenesis, to be associated with the age at onset in a large group of European Huntington disease patients. To confirm this association in a second independent patient cohort, we analysed the ATG7 V471A polymorphism in additional 1,464 European HD patients of the “REGISTRY” cohort from the European Huntington Disease Network (EHDN). In the entire REGISTRY cohort we could not confirm a modifying effect of the ATG7 V471A polymorphism. However, analysing a modifying effect of ATG7 in these REGISTRY patients and in patients of our previous HD cohort according to their ethnic origin, we identified a significant effect of the ATG7 V471A polymorphism on the HD age at onset only in the Italian population (327 patients). In these Italian patients, the polymorphism is associated with a 6-years earlier disease onset and thus seems to have an aggravating effect. We could specify the role of *ATG7* as a genetic modifier for HD particularly in the Italian population. This result affirms the modifying influence of the autophagic pathway on the course of HD, but also suggests population-specific modifying mechanisms in HD pathogenesis.

## Introduction

Huntington disease (HD) is one of the most common monogenetic neurodegenerative disorders and is clinically characterized by progressive development of motor disturbances as well as cognitive and psychiatric dysfunctions mainly starting at middle age [1]. The underlying genetic defect of HD is the expansion of an unstable CAG repeat in the *HTT* gene resulting in an elongated polyglutamine tract of the huntingtin protein (htt), which is inversely correlated with the age-at-onset (AAO) and the course of the disease [2]–[4]. The length of the polyglutamine tract accounts for 42 to 73% of the variance in the AAO [5], [6]. The remaining variance of AAO may be due to modifier genes and seems to be strongly heritable. However, environmental effects such as daily activity may also modify AAO in HD patients [7]. To date, several studies identified genetic modifiers of AAO in HD participating in glutamatergic transmission (*GRIK2, GRIN2A, GRIN2B*) [8]–[11], axonal trafficking (*HAP1*) [12], gene transcription (*TCERG1, TP53*) [13], [14], energy metabolism (*PPARGC1A*) [15]–[17] or protein degradation (*UCHL1, ATG7*) [18]–[20] representing various intracellular pathways involved in pathogenic processes of HD [21].

Recently, we found the V471A polymorphism in the *autophagy-related gene 7* (*ATG7*) to be associated with the AAO of HD in a large group of more than 900 European HD patients [20]. The gene product of *ATG7* is an important part in the autophagic machinery facilitating the degradation of long-lived proteins, protein complexes and damaged organelles in the cell [22]. Additionally, autophagy enables the degradation of aggregate-prone proteins such as mutant huntingtin (mhtt), which tends to form intracellular aggregates and fails to undergo proteasomal degradation [23], [24]. Our previous study revealed a significant effect of the ATG7 V471A polymorphism on the HD AAO leading to an approximately 4-years-earlier onset of the first symptoms [20]. The rare p.471A allele might affect ATG7 function and subsequently impairs the autophagic process and the degradation of mhtt. That ATG7 dysfunction could lead to neurodegeneration is supported by observations in ATG7-deficient mice, which exhibit a loss of cerebellar and cortical neurons and the formation of ubiquitin-positive aggregates [25], [26]. On the other hand, a general induction of autophagy results both in a reduction of soluble and aggregated mhtt and protects against mhtt-mediated toxicity in cell, fly and mouse models of HD [23], [27], [28].

In order to validate a true association of a genetic modifier with the AAO of a disease or to facilitate a more detailed association analysis, independent replication studies are mandatory. Therefore, we analysed the association of the ATG7 V471A polymorphism with the HD AAO, which we detected in a previous study, in a second independent population composed of more than 1,400 European HD patients and specified the modifying effect of ATG7 in different populations.

## Materials and Methods

### HD Patients

As the aim of this study is the analysis of the identified modifier polymorphism ATG7 V471A in a second HD patients cohort, we examined a patients group consisting of 1,464 unrelated European HD patients, which were obtained by the European Huntington’s Disease Network (EHDN) “REGISTRY” study prior to February, 2011 (2^nd^ European HD cohort = EHDN REGISTRY cohort). The EHDN REGISTRY project is a multicentre, prospective, observational study that enrols HD expansion carriers [29]. For all patients HD was clinically diagnosed. Motor, psychiatric and cognitive signs were scored using the Unified Huntington’s Disease Rating Scale (UHDRS) and AAO was estimated as the onset of motor symptoms. The mean AAO was 43.8 (SD 12.3) and ranged from 5 to 78 years. CAG repeat lengths in the *HD* gene of the patients were mainly examined by a PCR amplification followed by capillary electrophoresis by BioRep (Milan, Italy). The number of the expanded CAG repeats ranged from 37 to 89 with a median repeat number of 43. The EHDN REGISTRY cohort includes patients from different European countries except Germany and Italy.

Furthermore, we analysed additional patients of a 1^st^ European HD cohort that was already examined in a previous study [20]. This group consisted of a total of 943 European HD patients with subgroups of 371 patients of German and 327 patients of Italian descent. Compared to the number of investigated patients in the previous study [20] we managed it to re-genotype 25 HD patients of the 1^st^ European HD cohort, which failed during the first genotyping. Also for all these patients, HD was clinically diagnosed and AAO was estimated as the age at which motor symptoms first occurred. The mean AAO of the 1^st^ European cohort patients was 45.2 (SD 13.4) and ranged from 5 to 85 years. CAG repeat lengths of the expanded allele ranged from 39 to 90 units (median: 44). No overlap between the 1^st^ European HD cohort and the EHDN REGISTRY cohort is expected.

### Ethics Statement

The study was performed under a protocol approved by the Institutional Review Board of the University of Tuebingen Medical Faculty and the other sites of the EHDN REGISTRY project [29]. The participants gave informed written consent according to the International Conference on Harmonisation – Good Clinical Practice (ICH-GCP) guidelines (http://www.ich.org/LOB/media/MEDIA482.pdf) and according to the Declaration of Helsinki.

### Genotyping

As the polymorphism V471A in *ATG7* was already analysed in a large HD patient cohort in a previous study [20], we used the same genotyping conditions as described. So genotyping of ATG7 V471A (dbSNP rs36117895) was performed by standard PCR conditions and a following restriction analysis using 1U MboII according to manufacturer’s instructions (New England Biolabs Inc., Beverly, MA, USA). The nomenclature for numbering of changes at nucleotide or amino acid level follows general rules [30].

### Statistical Analysis

For a descriptive statistical analysis allele and genotype frequencies as well as Hardy-Weinberg distribution of the ATG7 V471A genotype was investigated by Genepop version 4.0.10 (http://genepop.curtin.edu.au/) (Table 1). Using the framework of linear models in an analysis of variance and covariance (JMP® Version 7.0.1, SAS Institute Inc., Cary, NC, USA), we tested the modifying role of the ATG*7* V471A polymorphism on the AAO of HD. First, we applied a model of analysis of variance with the ATG7 V471A polymorphism and the expanded HD allele as independent variables and the AAO as dependent variable. The goodness of fit was evaluated by the proportion of variation in the AAO explained by the coefficient of determination (R^2^). We obtained the best fit of our data and a minimization of the residuals by logarithmic transformation of the AAO and the CAG repeat number in the *HTT* gene. To determine the effect of the ATG*7* V471A polymorphism on AAO by an analysis of covariance in this model, the effect of the expanded CAG allele (HD CAG) was calculated alone, as well as with the V471A polymorphism. A change of R^2^ after adding the effect of the polymorphism indicated a relative improvement of the model and thus identified the percentage of variance that was attributable to the ATG7 V471A polymorphism when there was a significant P value (P≤0.05). Adjustment of multiple testing was performed according to Bonferroni correction. Differences in the AAO of HD within different genotypes were determined by a two-tailed *t* test (JMP® Version 7.0.1, SAS Institute Inc., Cary, NC, USA).

**Table 1:** Allele and genotype frequencies of ATG7 V471A polymorphism.

Group	Allele frequency^a^		Genotype frequency		
	V	A	VV	VA	AA
EHDN REGISTRY cohort (n = 1464)	0.961	0.039	0.947	0.053	0.000
1^st^ European cohort (n = 918)	0.960	0.040	0.919	0.081	0.000
Controls (n = 60)	0.967	0.033	0.933	0.067	0.000

The nomenclature for numbering of changes at nucleotide or amino acid level follows general rules [30].

The observed genotypes did not differ from expectations under Hardy-Weinberg equilibrium (EHDN REGISTRY cohort: P = 1.000; 1^st^ European cohort: P = 0.3920; controls: P = 1.000 [20]).

Genotype frequencies of Atg7 V471A in EHDN REGISTRY patients differ significantly from the respective frequencies in HD patients of the previous cohort (P = 0.0007).

n, number of investigated persons whose genotype could be determined.

a Allele frequency of nucleotide substitution in *ATG7* is described by V ( = valine) as major allele and A ( = alanine) as rare allele.

## Results

Analysing the ATG7 V471A polymorphism in patients of the EHDN REGISTRY [28] cohort as a second cohort of European HD patients and comparing them with patients of a previously examined 1^st^ European HD cohort [20] that does not overlap with the EHDN REGISTRY cohort, the respective alleles showed comparable frequencies. However, the heterozygous V471A genotype was significantly less frequent in the EHDN REGISTRY patients than in patients of the 1^st^ European HD cohort (P = 0.0007) (Table 1). The expanded CAG repeat in the *HTT* gene accounts for up to 66% of the variance in the AAO (R^2^ = 0.6633), so that about 34% of the AAO variance has to be determined by other factors acting as modifiers of the disease. In contrast to our previous study where we identified the ATG7 V471A polymorphism as a modifier of HD AAO, this polymorphism did not exert any overall significant influence on the AAO of the entire 1,464 EHDN REGISTRY patient cohort (Table 2). Similar results were obtained when analysing a potential effect of ATG7 V471A when grouping the patients according to longer and shorter CAG repeat lengths or sex (data not shown).

**Table 2:** Analysis of covariance of ATG7 in EHDN REGISTRY cohort.

Model	R^2^	ΔR^2^	P value	Least significant number of patients^a^
CAGexp	0.6633		<0.0001	6
HD CAG+Atg7 V471A	0.6633	0.0000	0.6151	22244

The level of significance was set to P = 0.05; n = 1464.

CAGexp, expanded CAG allele in huntingtin.

a Minimum number of patients, which are necessary to detect a significant effect of the analysed factor based on the respective genotypes.

In order to check whether the difference on the influence of the ATG7 V471A polymorphism on HD AAO in the two independent cohorts could be attributed to the origin of the patients, i.e. the different European countries, we first analysed the impact of the patients’ origin on their AAO. As this factor showed a highly significant effect on the HD AAO (P<0.0001), we split the HD patients of the EHDN REGISTRY cohort as well as the 1^st^ European HD cohort by ancestry and analysed the impact of the ATG7 V471A polymorphism in these single groups. Remarkably, the population specific analysis of an ATG7 V471A effect on HD AAO revealed an influence of the polymorphism in addition to the expanded HD allele only in Italian HD patients (P = 0.0119) (Table 3). In all other examined European populations ATG7 V471A did not exert any modifying effect on the disease onset. Also, the frequency of the heterozygous V471A genotype is significantly higher in Italian HD patients (P<0.05) than in the other populations, which could explain the higher heterozygosity rate in the 1^st^ European HD cohort of our previous study (Table 1). The higher heterozygosity rate in Italian HD patients may have provided sufficient power to detect the effect of the ATG7 V471A polymorphism on the HD AAO in this population as other analysed populations, which are comparable in size (e.g. patients from the UK), have a significantly less amount of patients with a heterozygous V471A genotype.

**Table 3:** Allele and genotype frequencies, Hardy-Weinberg distribution and analysis of covariance of ATG7 V471A in different European populations.

Country	Allele frequency^a^		Genotype frequency			Hardy-Weinberg	ANOVA^c^ P-value
	V	A	VV	VA	AA		
Denmark (n = 101)	0.960	0.040	0.921	0.079	0.000	1.000	0.7797
France (n = 134)	0.981	0.019	0.963	0.037	0.000	1.000	0.8140
Netherlands (n = 174)	0.983	0.017	0.966	0.034	0.000	1.000	0.5537
Poland (n = 242)	0.988	0.012	0.975	0.025	0.000	1.000	0.3862
Spain (n = 168)	0.970	0.030	0.940	0.060	0.000	1.000	0.1481
UK (n = 347)	0.970	0.030	0.939	0.061	0.000	1.000	0.2611
Germany (n = 371)^b^	0.958	0.042	0.917	0.083	0.000	1.000	0.2988
Italy (n = 327)	0.946	0.054	0.893	0.107	0.000	0.6122	0.0238*

Populations with ≥100 examined HD patients are shown; *n* number of investigated HD patients.

a Allele frequency of nucleotide substitution in *ATG7* is described by V ( = valine) as major allele and A ( = alanine) as rare allele.

b Compared to the number of investigated patients in the previous study [20] we managed it to re-genotype 25 HD patients of the 1st European HD cohort, which failed during the first genotyping.

c Analysis of covariance showing the effect of the ATG7 V471A polymorphism on the HD AAO in the respective population.

* significant effect, the level of significance was set to P = 0.05 and the p-value was adjusted for multiple testing according to Bonferroni correction.

Thereby, the heterozygous V471A genotype in Italian HD patients, who develop their first symptoms at an average age of 42.4 years, is associated with an approximately 6-years earlier onset of the disease compared to patients homozygous for the major allele (Table 4).

**Table 4:** Mean ages-at-onset of the different Atg7 genotypes in Italian HD patients.

Genotype	Number of patients (n = 327)	Mean CAGexp (SD)	Mean AAO (SD)
VV	292	45.47 (4.73)	48.12 (13.87)^a^
VA	35	46.31 (7.66)	42.43 (14.68)
AA	–	–	–

CAGexp, expanded CAG repeat number in huntingtin, SD standard deviation, V major allele valine at amino acid position 471 (V471), A rare allele alanine at amino acid position 471 (A471).

a t test: patients with genotype VV differ significantly from patients with heterozygous VA genotype (P = 0.0348).

Interestingly, Italian HD patients have the oldest mean AAO compared with patients from the other European countries analysed in this study, which differs significantly from the mean AAO of other European patients (Table 5). HD patients from Denmark and France show a comparatively similar AAO (P≥0.05), which is not modified by the genotype of the Atg7 V471A polymorphism.

**Table 5:** Mean ages at onset and CAG repeat numbers of the expanded huntingtin allele in different European populations.

Country	Mean AAO (SD)				Mean CAGexp (SD)		
	Atg7 genotype		all patients	T-test P-value a	Atg7 genotype		all patients
	VV	VA			VV	VA	
Denmark (n = 101)	46.53 (11.85)	46.13 (7.24)	46.50 (11.53)	n.s.	43.62 (4.70)	43.38 (2.07)	43.6 (4.51)
France (n = 134)	46.19 (12.09)	38.00 (11.81)	45.89 (12.14)	n.s.	44.18 (3.47)	47.00 (4.06)	44.28 (3.51)
Netherlands (n = 174)	44.42 (11.57)	46.00 (11.28)	44.45 (11.53)	n.s.	43.67 (3.12)	42.17 (1.17)	43.61 (3.08)
Poland (n = 242)	40.25 (13.93)	40.83 (15.54)	40.26 (13.93)	n.s.	46.11 (7.31)	44.50 (3.83)	46.07 (7.24)
Spain (n = 168)	43.04 (11.89)	51.10 (17.08)	43.52 (12.34)	n.s.	44.73 (3.98)	43.80 (5.03)	44.67 (4.04)
UK (n = 347)	44.93 (11.68)	42.19 (13.15)	44.76 (11.77)	n.s.	44.14 (3.69)	44.62 (3.57)	44.17 (3.68)
Germany (n = 371)	44.10 (12.77)	42.42 (14.74)	44.06 (12.90)	n.s.	45.61 (4.80)	45.90 (4.21)	45.61 (4.71)
Italy (n = 327)	48.12 (13.87)	42.43 (14.68)	47.52 (13.95)^b^	0.0348	45.47 (4.73)	46.31 (7.66)	45.56 (5.04)

Populations with ≥100 examined HD patients are shown; *n* number of investigated HD patients.

AAO, age at onset, CAGexp, expanded CAG repeat number in huntingtin, SD standard deviation, V major allele valine at amino acid position 471 (V471), A rare allele alanine at amino acid position 471 (A471).

a t test: Comparing the mean AAO of the VV and VA genotypes in the different populations, only Italian patients with genotype VV differ significantly from patients with heterozygous VA genotype (P = 0.0348).

b t-test: Italian patients show the oldest mean AAO, which is significantly older than the mean AAO of patients from the other presented European countries together (Italy vs other countries: P<0.0001), but not significantly different from the mean AAO of patients from Denmark (P = 0.4728) and France (P = 0.2050).

In the Italian population, the expanded HTT allele itself accounts for ∼46% (R^2^ = 0.4595) of the variance in the HD AAO, thus allowing a broader range for an additional influence of disease modifying factors. The ATG7 V471 polymorphism contributes ∼1.7% to the variance in the AAO representing ∼3% of the variance that cannot be accounted for by the expanded CAG repeat in the *HTT* gene. Interestingly, it can be observed that both in patients of the Italian population separately and in the whole EHDN REGISTRY cohort, the rare p.471A allele is, with the exception of one Italian patient, only present in patients with shorter HD alleles with ≤55 CAG units. Thus, the rare p.471A allele seems to assert its modifying effect in conditions with shorter and thus less severely affecting CAG expansions that potentially allow a greater influence of additional modifiers.

## Discussion

The present study intended to confirm the modifying effect of the V471A polymorphism in the *ATG7* gene, previously identified in more than 900 European HD patients, in a second independent cohort of HD patients. Instead of a replication of our previous results in this second cohort of more than 1400 European HD patients, which is expected to have no overlap with the patients of our previous study, we could only detect the modifying effect of the ATG7 V471A polymorphism in Italian HD patients of our 1^st^ European HD cohort. Here, the rare p.471A allele of the polymorphism is associated with a 6-years earlier onset of the first symptoms and thus potentially has an aggravating effect on the disease.

With this result, we had to revise and modify the finding of our previous study where we originally did not observe a population specific effect of the ATG7 V471A polymorphism [20]. Re-genotyping and addition of more patients to the analysis of the 1^st^ European HD cohort lead to an exclusion of the ATG7 V471A influence on HD AAO in the German patients. This highlights the importance that the size of a collective needs to be as big as possible to indicate a real effect of a potential modifier.

In the analysed Italian population the modifying influence of the ATG7 V471A polymorphism seems to be robust. It represents about 3% of the variance in the AAO that cannot be accounted for by the expanded CAG repeat in the *HTT* gene itself. The CAG repeat determines about 46% of the whole variance in the HD AAO and thus is in accordance with a former study that analysed the CAG repeat expansion in Italian HD families [31]. In comparison with different studies, the CAG repeat in the Italian population seems to have a relative weaker effect on the AAO [5], [6], [32] and additional factors may have a greater impact on the AAO. Depending on the population, the length of the CAG tract accounts for up to 73% of the variance in the AAO [5], [6], [32]. The remaining variance in the AAO, which is not accounted for by the CAG repeat, is determined by other genetic and environmental factors [33]. The impact of these additional factors also varies in different populations. Sibling analyses revealed a contribution of familial (genetic) factors to the HD AAO variance of up to 19% in addition to the expanded CAG repeat [34], [35], but as a genetic factor modifies the HD AAO in one population, it can have less or even no influence in a second population. Examples of this are polymorphisms in the genes encoding the brain-derived neurotrophic factor (BDNF) [36]–[38], the glutamate receptor GRIK2 [8], [9], [39]–[41] or the CAG repeat length of the normal huntingtin allele [39], [42]–[45]. Such a population-specific effect of disease modifying factors is also seen in other neurodegenerative diseases like Parkinson disease (PD) or Alzheimer disease. In this regard, BDNF shows a modifying effect in a cohort of Greek and Italian PD patients [46], [47], but not in Finnish or Swedish patients [48], [49].

The existence of population-specific effects of genetic modifiers in HD is supported by the identification of potential chromosomal modifier loci, which were achieved by genome wide linkage analyses in different populations. The so called HD MAPS study, which analysed HD patients mainly from the United States and Canada, identified potential modifier loci on the chromosomal regions 4p16, 6p21–23, 6q23–26 and 18q22 [50], [51]. A linkage scan in Venezuelan HD kindreds revealed loci at 2p25 and 2q35 as regions that harbour potential modifier genes, but could not confirm locus 18q22 of the HD MAPS study [52]. The chromosomal locus of *ATG7* itself is on chromosome 3p25.3 and thus the gene is not located in any potential modifier region identified so far. It remains to be seen whether this region could be identified as a linkage locus when analysing HD patients particularly from Italy or Southern Europe.

Notably, a population specific effect of genetic modifiers on the HD AAO has been recently reported in the Italian population for the mitochondrial regulator PGC-1α [53]. We and others have observed that specific *PPARGC1A* SNPs are associated with the AAO of HD symptoms [15]–[17]. The modifying effect was mainly observable in Italian HD patients [16], [17], [53] and Ramos and coworkers suggested that this could be attributed to a population-dependent phenotype stratification as HD patients from Italy and Southern Europe were found to have a significantly older mean AAO than patients from other European regions [53]. Indeed, the Italian HD patients of the present study also showed a significantly older AAO than patients from most other European countries, although the sizes of the expanded CAG repeats are similar. While HD patients from France or Spain also show the tendency, though not significant, for a marked difference in the AAO of ATG7 V471A genotypes compared to Italian HD patients their mean AAO is in the general AAO range of most other European populations. A later AAO, particularly in Southern Europe, may be due to further genetic factors, which modify the course of HD only in Southern European populations, but are not present in HD patients from other European regions. However, a later AAO may also reflect population differences, which could be due to genetic and/or environmental factors (such as method of diagnosis and lifestyle or nutrition) as were already discussed by Ramos and coworkers [53]. It is essential to consider such additional environmental factors in future investigations. However, examining the Italian patients of our study as a cohesive group, the method of diagnosing HD as well as other population specific environmental factors should be comparable. So, it may be possible that the ATG7 V471A polymorphism acts as a true genetic modifier in the Italian population. Most likely it seems to be in linkage disequilibrium with another genetic variation, potentially located in ATG7 itself. In this regard, the current study may also direct future modifier AAO studies in HD, such as more population stratification, stronger selection based on a more clearly defined AAO definition and even on selection of patients with a specific range of expanded polyQ length.

In conclusion, the findings presented here confirm a modifying role of the ATG7 V471A polymorphism on the AAO of HD, but with a specific effect in the Italian population. Despite this limitation, they provide further indication for a crucial link between autophagy and HD pathogenesis. However, further studies on the functional role of this potential genetic modifier are required to establish its role in HD pathogenesis and to potentially guide therapeutic approaches.
